# Supplementary figures and images for: Selection of internal references for RT-qPCR assays in Neurofibromatosis type 1 (NF1) related Schwann cell lines
Source: PLoS One. 2021 Feb 25;16(2):e0241821. doi: 10.1371/journal.pone.0241821 (PMC7906369; doi:10.1371/journal.pone.0241821)

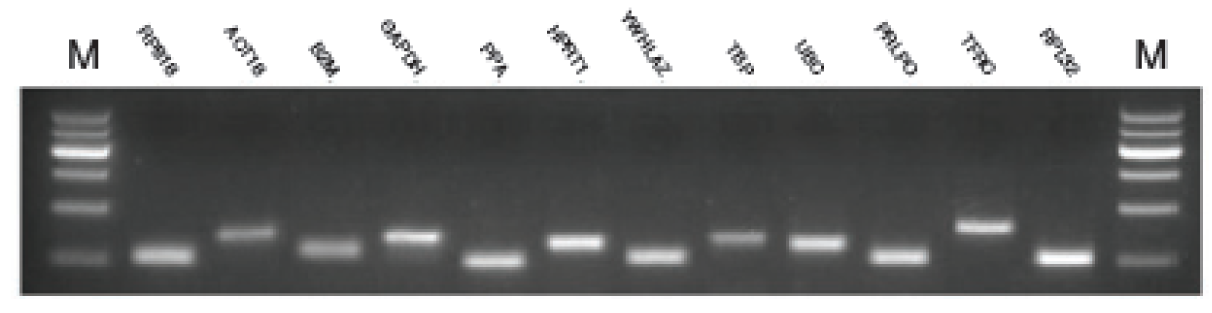

Supplement: S1 Fig — Agarose gel electrophoresis of RT-qPCR amplification products for each of the internal reference genes provided by Sangon (M: Sangon DNA Marker {B600335}). (TIFF) [file pone.0241821.s002.tiff]
