# Supplementary material for: Selection of internal references for RT-qPCR assays in Neurofibromatosis type 1 (NF1) related Schwann cell lines
Source: PLoS One. 2021 Feb 25;16(2):e0241821. doi: 10.1371/journal.pone.0241821 (PMC7906369; doi:10.1371/journal.pone.0241821)
Supplement: S3 Table — (PDF) [file pone.0241821.s005.pdf]

S3 Table. Primer list

Gene symbol	Order NO.	Amplicon length (bp)	Position	NCBI Accession No.	Full name
Rps18	B661101	106	exton2/3	NM_022551	18S Ribosomal RNA
ACTB	B661102	144	exton5/6	NM_001101	β-actin
B2M	B661103	114	exton2/3	NM_004048	β-2-microglobulin
GAPDH	B661104	138	Exton6/7	NM_002046	Glyceraldehyde-3-phosphate dehydrogenase
PPIA	B661105	97	exton1/3	NM_021130	Peptidylprolyl isomerase A
HPRT1	B661106	131	exton1/2	NM_000194	Hypoxanthine phosphoribosyltransferase 1
TBP	B661108	132	exton5/6	NM_003194	TATA binding protein
UBC	B661109	123	exton1/2	NM_021009	Ubiquitin C
RPLP0	B661110	101	exton2/3	NM_001002	Ribosomal protein large P0
TFRC	B661111	156	exton2/3	NM_003234	Transferrin receptor (p90, CD71)
RPL32	B661112	100	exton3/4	NM_000994	Ribosomal protein L32

* All the primers were commercial products validated by Sangon Biotech (Shanghai, China)
